# Supplementary material for: P2Y1 and P2Y12 Receptors Mediate Aggregation of Dog and Cat Platelets: A Comparison to Human Platelets
Source: Int J Mol Sci. 2025 Jan 30;26(3):1206. doi: 10.3390/ijms26031206 (PMC11818226; doi:10.3390/ijms26031206)
Supplement: Supplementary file 1 [file ijms-26-01206-s001.zip › ijms-3378032-supplementary.pdf]

International Journal of
Molecular Sciences

Article

Supplementary Files: P2Y1 and P2Y12 Receptors Mediate Aggregation of Dog and Cat Platelets: A Comparison to Human Platelets

Reece A. Sophocleous [1], Stephen J. Curtis [2], Belinda L. Curtis [2], Lezanne Ooi [1] and Ronald Sluyter [1*]

[1] Molecular Horizons and School of Chemistry and Molecular Bioscience, University of Wollongong, Wollongong, NSW, 2522, Australia; reece.sophocleous@sydney.edu.au (R.A.S.); lezanne@uow.edu.au (L.O.)
[2] Your Village Vet Balgownie, Balgownie, NSW, 2519, Australia; stephen.curtis@yourvillagevet.com.au (S.J.C); belinda.curtis@yourvillagevet.com.au (B.L.C)
* Correspondence: rsluyter@uow.edu.au (R.S); Tel.: +61-2-4221-5508

Supplementary Data

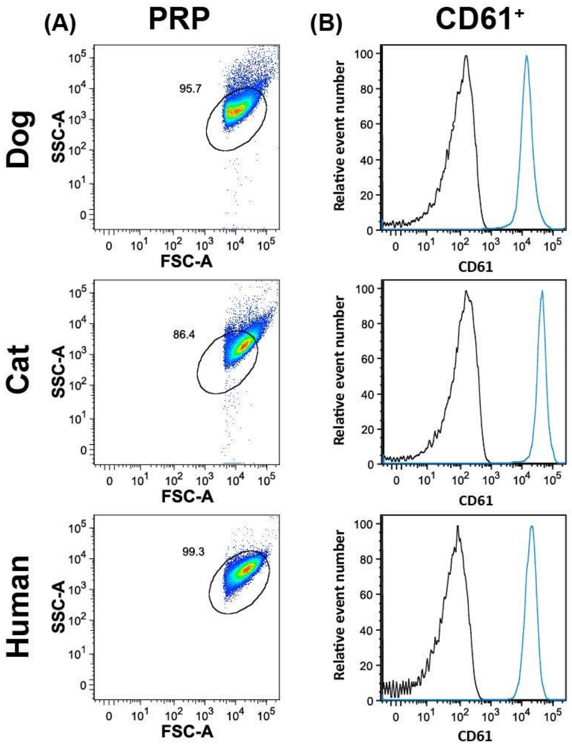

Supplementary Figure 1. Flow cytometry gating of dog, cat and human platelets. (A, B) PRP samples isolated from dog, cat, or human blood were labelled with APC-conjugated anti-CD61 or isotype control antibody and the proportions of platelets were determined by flow cytometry. (A) Forward scatter area (FSC-A) and side scatter area (SSC-A) were used to identify and subsequently analyse platelets in dog (top panels), cat (middle panels) or human (bottom panels) PRP. (B) Platelets from dogs, cats, or humans labelled with APC-conjugated anti-CD61 (blue histogram) or isotype control (black histogram) antibody. Data are representative of three independent experiments.

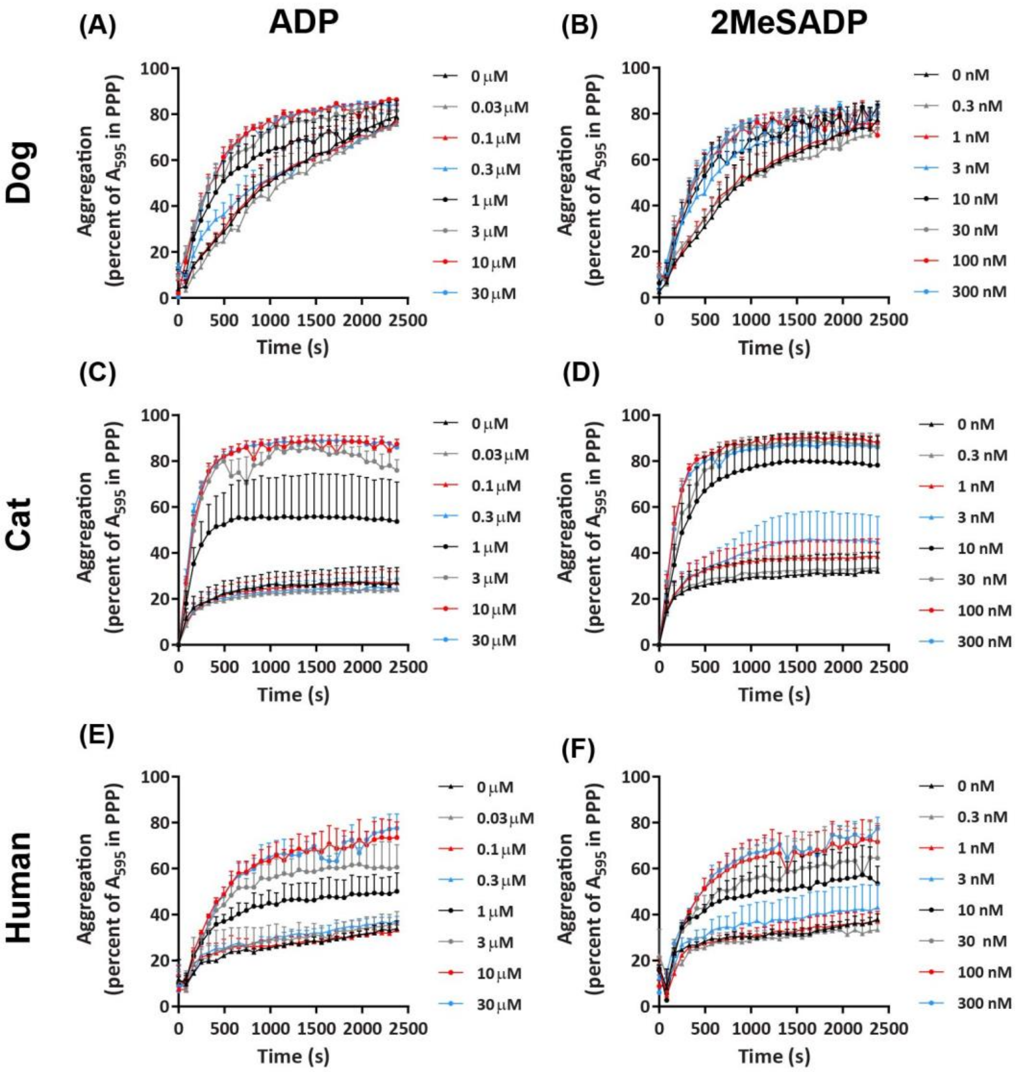

Supplementary Figure 2. ADP- and 2MeSADP-induced platelet aggregation in dogs, cats and humans over time. PRP and PPP, isolated from (A, B) dog, (C, D) cat or (E, F) human whole blood, (were incubated in the absence (Tyrode's buffer) or presence of increasing concentrations ADP or 2MeSADP and platelet aggregation was measured over 45 minutes. Percent of aggregation for each PRP sample was calculated relative to the percent of aggregation in the corresponding PPP sample at each time point. Data shown for each species are mean ± SEM from three independent experiments.
